# Can Reptile Embryos Influence Their Own Rates of Heating and Cooling?

**DOI:** 10.1371/journal.pone.0067095

**Published:** 2013-06-24

**Authors:** Wei-Guo Du, Ming-Chung Tu, Rajkumar S. Radder, Richard Shine

**Affiliations:** 1 School of Biological Sciences, University of Sydney, Sydney, New South Wales, Australia; 2 Key Lab of Animal Ecology and Conservation Biology, Institute of Zoology, Chinese Academy of Sciences, Beijing, People’s Republic of China; 3 Department of Life Science, National Taiwan Normal University, Taipei, Taiwan; St. Joseph's Hospital and Medical Center, United States of America

## Abstract

Previous investigations have assumed that embryos lack the capacity of physiological thermoregulation until they are large enough for their own metabolic heat production to influence nest temperatures. Contrary to intuition, reptile embryos may be capable of physiological thermoregulation. In our experiments, egg-sized objects (dead or infertile eggs, water-filled balloons, glass jars) cooled down more rapidly than they heated up, whereas live snake eggs heated more rapidly than they cooled. In a nest with diel thermal fluctuations, that hysteresis could increase the embryo’s effective incubation temperature. The mechanisms for controlling rates of thermal exchange are unclear, but may involve facultative adjustment of blood flow. Heart rates of snake embryos were higher during cooling than during heating, the opposite pattern to that seen in adult reptiles. Our data challenge the view of reptile eggs as thermally passive, and suggest that embryos of reptile species with large eggs can influence their own rates of heating and cooling.

## Introduction

Many physiological processes are sensitive to temperature, placing a selective premium on the ability of organisms to maintain body temperatures within optimal ranges [1]. Since the 1960’s, we have known that terrestrial reptiles control their body temperatures not only behaviourally (e.g. sun-basking [2]) but also physiologically: many reptiles heat faster than they cool (hysteresis), by adjusting heart rates and blood flow [3], [4]. Nonetheless, the small size and relatively undeveloped physiology of embryonic reptiles have encouraged the assumption that embryos cannot influence their own body temperatures [5], [6]. That assumption was falsified by recent experimental evidence of behavioural thermoregulation: turtle embryos move to exploit subtle thermal gradients within the egg [7]. Can reptile embryos thermoregulate physiologically as well as behaviourally, like free-living reptiles?

Previous investigations have assumed that embryos lack the capacity of physiological thermoregulation until they are large enough for their own metabolic heat production to influence nest temperatures [8]. How robust is this conclusion? Certainly, an embryo cannot generate enough metabolic heat to affect its temperature until it is very large (close to hatching [9]). However, an embryo might modify its rates of thermal exchange with the outside world by (a) control over water exchange (and thus, heat exchange) between the egg’s surface and the environment [10]; and/or (b) changing heart rates, to modify thermal exchange via blood circulation [11]. If embryos can influence such processes, the incubation environment may be partly under embryonic control.

What kinds of embryos might be under strong selection to influence their own temperatures? Among terrestrial vertebrates, reptiles offer a better model than do birds, because in most reptiles (a) incubation temperatures influence hatching success, developmental rates and hatchling phenotypic traits, and thus fitness [12], and (b) eggs are not attended by the female, or are attended only intermittently [13], [14], thereby reducing the degree of parental thermal control. Most reptile eggs are small, and thus heat and cool quickly [4]. Large eggs provide the best opportunity to detect embryonic control over thermal exchange rates, however, because slower heating and cooling provides a greater potential selective advantage to modifying such rates, and facilitates detection of thermal hysteresis. Accordingly, we examined the large eggs of the Indian Python (*Python molurus*), the Australian Carpet Python (*Morelia spilota*) and the stripe-tailed ratsnake (*Elaphe taeniura*), to see if snake embryos are able to influence the rates at which they heat and cool.

## Results

**Figure 1:**
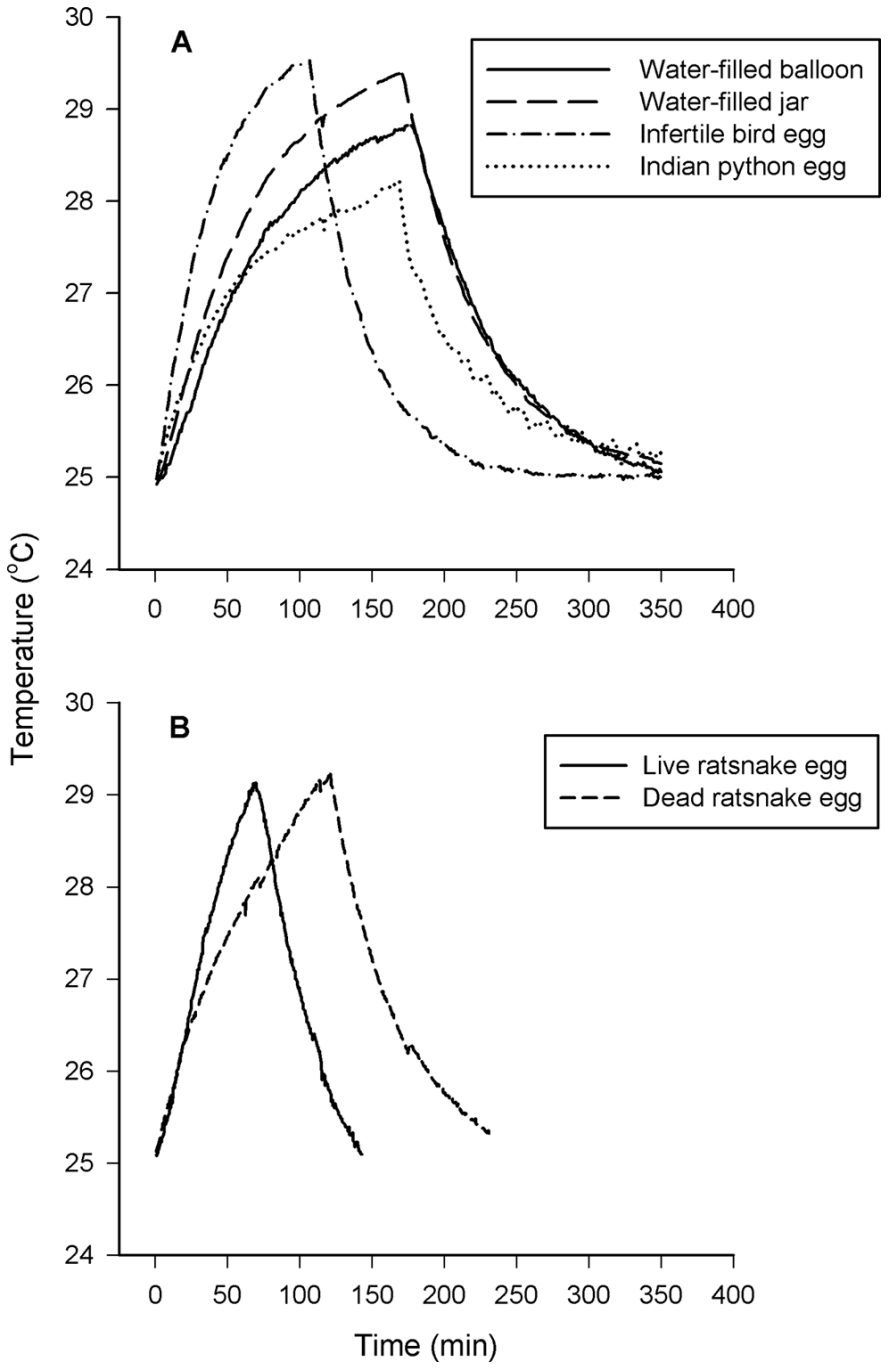
Temperature changes of live reptile eggs and dead or inanimate controls when they were heated and cooled. (a) The large eggs of Indian pythons took longer to heat and cool than did infertile bird eggs and inanimate objects such as water-filled balloons and jars. (b) Live eggs of ratsnakes heated faster than did dead eggs. The graphs show mean temperatures of eggs and inanimate controls; error bars are omitted to avoid cluttering the Figure.

As expected from their larger size, the eggs of pythons took longer to heat or cool than did the eggs of ratsnakes or birds, or inanimate objects such as water-filled balloons and jars (Fig. 1A,B). Live eggs of ratsnakes heated faster than did dead eggs of the same species (Fig. 1B).

**Figure 2:**
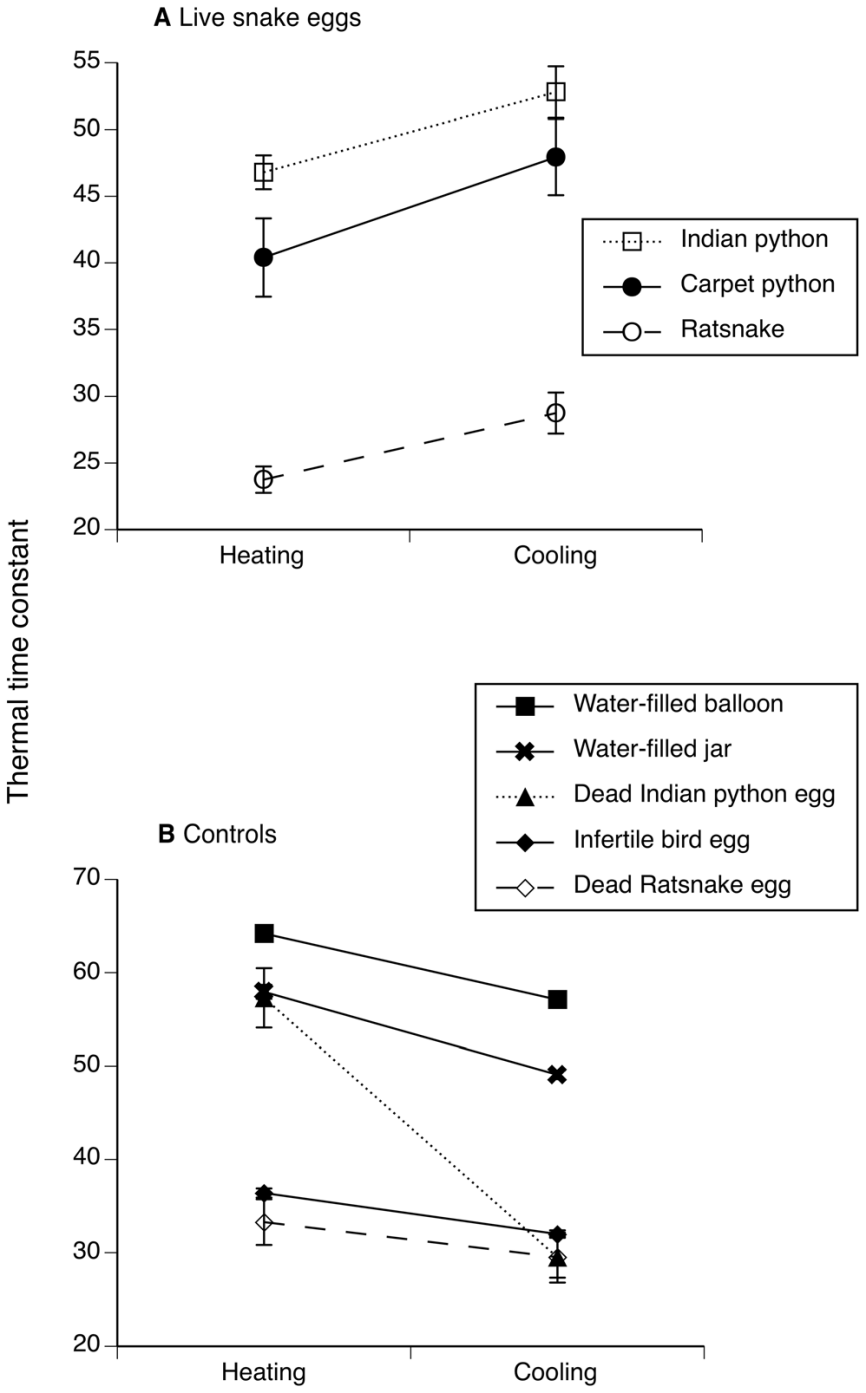
Rates of heating and cooling in live reptile eggs compared to dead or inanimate controls. (a) Live eggs of three snake species (Indian python, Carpet python and Chinese ratsnake) heated more rapidly than they cooled. (b) Controls either heated and cooled at similar rates, or heated more slowly than they cooled (a higher thermal time constant means slower rates of thermal change). Data are shown as mean ± SEM.

Live eggs heated faster than they cooled, in all three species that we tested (comparing thermal time constants: Indian python, paired *t*_15_ = 3.71, *p = *0.002; carpet python, paired *t*_8_ = 2.37, *p = *0.04; ratsnake, paired *t*_11_ = 4.09, *p = *0.001: Fig. 2A). In contrast, the dead eggs and inanimate objects that we tested either heated and cooled at the same rate (dead eggs of ratsnakes, paired *t*_11_ = 1.12, *p* = 0.28) or heated more slowly than they cooled (dead eggs of Indian pythons, paired *t*_2_ *= *11.39, *p* = 0.007; infertile chicken eggs, paired *t*_19_ = 8.86, *p = *0.0001; water-filled jars, paired *t*_9_ = 12.06, *p = *0.0001; water-filled balloons, paired *t*_18_ = 11.28, *p = *0.001: Fig. 2B).

**Figure 3:**
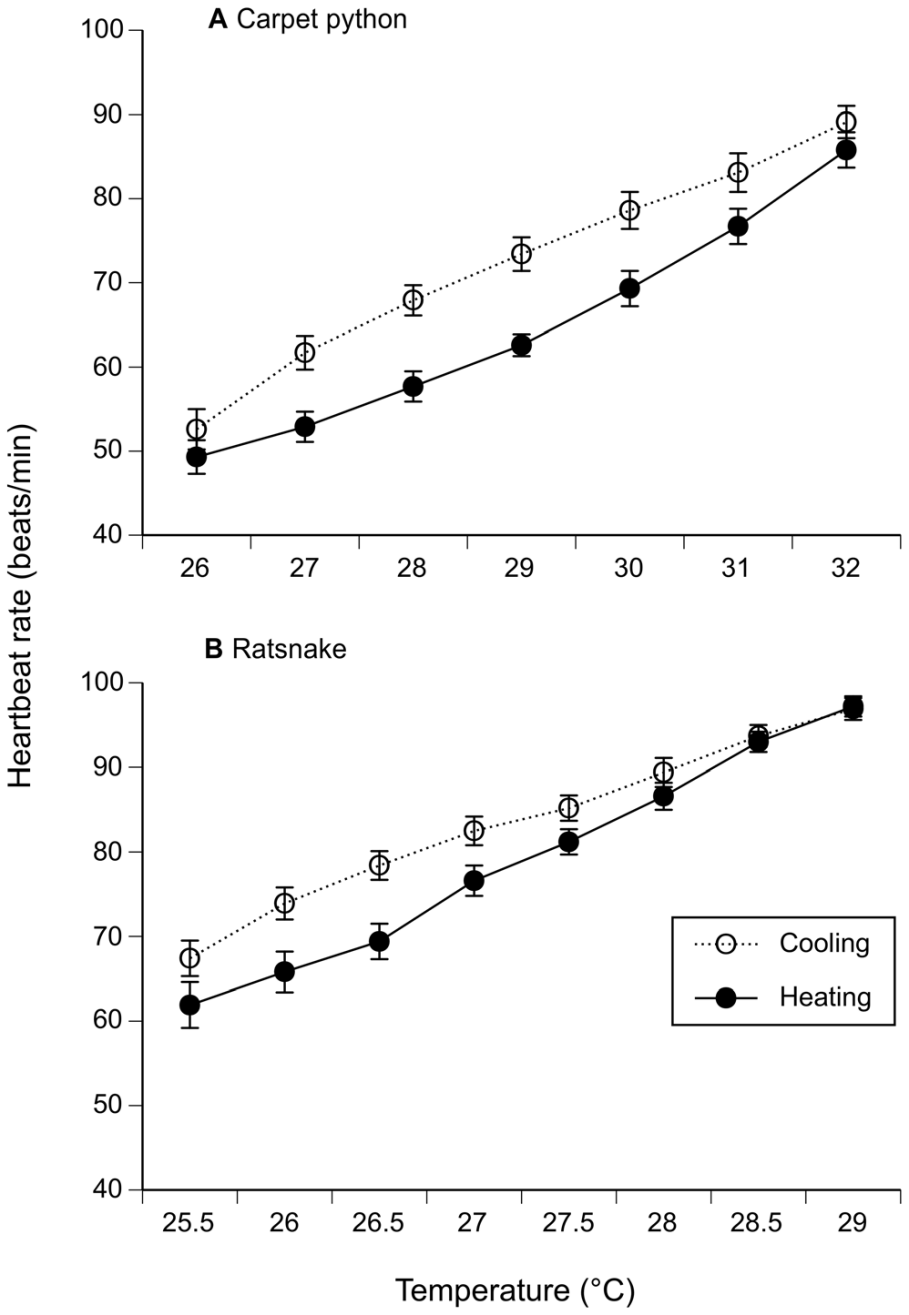
Hysteresis in heart rates of embryonic snakes. (a) Carpet pythons and (b) Chinese ratsnakes) exposed to rising or falling temperatures. Temperatures were monitored by thermocouples beside the embryo. At the same test temperature, heart rates were higher during cooling than during heating (mean beats per minute ± SEM).

Embryonic heart rates increased at higher temperatures. At the same temperature, heart rates were higher during cooling than heating (carpet python *F*_1,56_ = 66.53, *p*<0.0001; ratsnake *F*_1,88_ = 95.54, *p*<0.0001: Fig. 3).

## Discussion

Our results suggest that embryos are active participants in ecological processes, not simply organisms-in-progress. The phenotypes of hatchling reptiles are sensitive to minor thermal fluctuations during incubation, suggesting that thermoregulatory ability could enhance fitness [15]. In support of this idea, turtle embryos reposition themselves within the egg to exploit small thermal gradients [7]. Thus, the only question is feasibility: can a tiny embryo really influence the rate that it heats and cools? Contrary to intuition, our study suggests significant embryonic control over rates of thermal exchange, at least in relatively large eggs.

Most reptiles lay their eggs in damp nests, because permeable-shelled eggs rely on water exchange with the substrate [16]. Heat is exchanged between the egg and the environment through the shell, part of which is in contact with the air, and part in contact with the substrate. The rate of heat exchange is 23 times greater in water than in air [17] and so, heat exchange will proceed rapidly (per cm^2^ of egg surface) through the parts of the shell in contact with the (evaporating, and thus cool) damp substrate. Thus, an egg in contact with the damp substrate will tend to cool down more rapidly than it heats up (unless the egg chamber is fully saturated).

How do live snake eggs reverse this effect, to heat faster than they cool (Fig. 2A)? We doubt that embryos could reposition themselves [7] rapidly enough to influence their exposure to local temperatures. Cardiovascular flexibility may play a critical role, as it does in adult reptiles [4], [14], [18]. Our data reveal that hysteresis in heart rates during heating versus cooling occurs in embryonic reptiles, but in the opposite direction to that seen in adults. Adult heart rates are higher during heating than cooling, allowing sun-warmed blood in superficial vessels to be deployed to functionally important parts of the animal’s body [3], [18]. But, why do embryos (unlike adults) exhibit higher heart rates during cooling not heating? We do not pretend to understand how decreased heartbeat rates during heating could enhance rather than retard heat uptake, except to note that redistribution of vascular flow could influence water and heat exchange across the eggshell [14].

Thermoregulatory abilities may begin very early in a reptile’s life: like an adult, an embryonic reptile may be able to influence its own temperatures both by behaviour [7] and physiology (current study). In turn, that ability may affect developmental rates and thermally-dependent phenotypes (e.g., in species with temperature-dependent sex determination [19]), highlighting the importance of behavioral and physiological responses in understanding the effects of climate change on conservation of these species. For many reptile species with small eggs incubating in moist soil, rates of heat exchange likely will be too high for hysteresis to have any significant effect on overall incubation regimes. For species with large eggs, however, embryonic control over thermal parameters may be biologically significant. Even in species where females brood the clutch and maintain high stable incubation temperatures via shivering thermogenesis, incubation temperatures are far from stable (reflecting intermittent cessation of maternal brooding [20], [21]). Future research, especially in the field, could usefully explore how the attributes of eggs (e.g., size) and nests (e.g., humidity, substrate, maternal attendance) influence an embryo’s opportunities for physiological control of its own temperature. Extensive research on thermoregulation in the free-living stages of the reptile life-history provides a wealth of approaches and concepts that could usefully be applied to the thermal biology of embryonic reptiles.

## Materials and Methods

### (a) Study Species and Egg Incubation

We obtained captive-laid eggs from snakes of three species: *Elaphe taeniura* (stripe-tailed ratsnake, *n* = 12 eggs), *Morelia spilota* (Australian carpet python, *n* = 9 eggs) and *Python molurus* (Indian python, *n* = 16 eggs). Ratsnakes were sourced from a private farm in southern China, and the pythons from the Australian Reptile Park. The eggs were incubated on moist vermiculite (−200 kPa) at 30°C, and we tested their heating and cooling rates two-thirds through the incubation period (45 of 65 days, mean egg mass 203.3 g for Indian pythons; 42 of 60 days, mean egg mass 45.0 g for carpet pythons; 36 of 54 days, mean egg mass 25.2 g for ratsnakes). At this stage of incubation, embryos are well-differentiated but still relatively small (about 25% the mass of full-term embryos [22]).

### (b) Experimental Procedures

Eggs were incubated in open-topped 16×10×6 cm plastic boxes containing damp vermiculite (−200 kPa) weighing half as much as the egg (to mimic the damp substrates of natural nests). The eggs were heated and cooled between 25 and 30°C by shifting them between two different incubators set at the low and high temperatures. Three to five eggs were monitored per trial; order of heating vs. cooling was randomised. Heating and cooling rates were quantified as thermal time constants (τ), by regressing ln(T_b_ – T_e_) on time (T_b_ = body temperature, T_e_ = equilibrium temperature; the slope of this regression is equal to −1/τ) [23]. Airflow rates above the focal objects averaged 0.2 km/hr. For the eggs of carpet pythons and ratsnakes, we also measured heart rates of embryos non-invasively by infrared detectors (Buddy Digital Egg Monitor: Avian Biotech, Cornwall, UK [24]), as eggs heated and cooled over the thermal range 25 to 32°C.

To compare thermal time constants of live snake eggs to those of similar-sized inanimate or dead objects, we also monitored heating and cooling rates of physical models [water-filled balloons and glass jars (200 ml capacity, mean mass = 198.8 g; *n* = 19 and 10 respectively)], dead eggs of Indian pythons and ratsnakes (*n* = 3, 12 respectively) and infertile eggs of domestic chickens (*n* = 20). Internal temperatures of both live and dead eggs as well as physical models were monitored every minute using 40 gauge thermocouples (TCTTT140: Temperature Controls Pty Ltd., Sefton, Australia) connected to a data-taker (DT-80: Datataker, Scoresby, Australia). We localised the embryo within each egg by inspection under a light source, then inserted the thermocouple near the embryo. Paraffin wax sealed the puncture to prevent moisture loss. Puncturing and thermocouple insertion did not affect embryo survival, and all eggs continued to develop successfully after our experiments concluded.

All experimental procedures were approved by the Animal Care and Ethics Committee at the University of Sydney (L04/7-2007/3/4665), and were conducted in accordance with the NIH *Guide for the Principles of Animal Care*. The owners of Australian Reptile Park (Mr. John Weigel), and the private farm in southern China (Mr. Yan Zhang) permitted us to collect snake eggs from their snake ranches. As the species used in this study are not endangered or protected, no specific permissions are needed according to the current laws of China.

### (c) Statistical Analyses

Paired *t*-tests were used to compare thermal time constants between warming versus cooling (both in live eggs and in controls), and a repeated-measures ANOVA was performed to examine differences in heart rates during warming versus cooling.
